# Biophysical comparison of four silver nanoparticles coatings using microscopy, hyperspectral imaging and flow cytometry

**DOI:** 10.1371/journal.pone.0219078

**Published:** 2019-07-31

**Authors:** Robert M. Zucker, Jayna Ortenzio, Laura L. Degn, Jeremy M. Lerner, William K. Boyes

**Affiliations:** 1 U.S. Environmental Protection Agency, Office of Research and Development, National Health and Environmental Effects Research Laboratory, Toxicology Assessment Division, Research Triangle Park, NC, United States of America; 2 Oak Ridge Institute for Science and Education (ORISE) appointee at the National Health and Environmental Effects Research Laboratory, USEPA, RTP, NC, United States of America; 3 USEPA, RTP, NC, United States of America; 4 LightForm, Inc, NC, United States of America; Aarhus University, DENMARK

## Abstract

This study compared the relative cellular uptake of 80 nm silver nanoparticles (AgNP) with four different coatings including: branched polyethyleneimine (bPEI), citrate (CIT), polyvinylpyrrolidone (PVP), and polyethylene glycol (PEG). A gold nanoparticle PVP was also compared to the silver nanoparticles. Biophysical parameters of cellular uptake and effects included flow cytometry side scatter (SSC) intensity, nuclear light scatter, cell cycle distributions, surface plasmonic resonance (SPR), fluorescence microscopy of mitochondrial gross structure, and darkfield hyperspectral imaging. The AgNP-bPEI were positively charged and entered cells at a higher rate than the negatively or neutrally charged particles. The AgNP-bPEI were toxic to the cells at lower doses than the other coatings which resulted in mitochondria being transformed from a normal string-like appearance to small round beaded structures. Hyperspectral imaging showed that AgNP-bPEI and AgNP-CIT agglomerated in the cells and on the slides, which was evident by longer spectral wavelengths of scattered light compared to AgNP-PEG and AgNP-PVP particles. In unfixed cells, AgNP-CIT and AgNP-bPEI had higher SPR than either AgNP-PEG or AgNP-PVP particles, presumably due to greater intracellular agglomeration. After 24 hr. incubation with AgNP-bPEI, there was a dose-dependent decrease in the G_1_ phase and an increase in the G_2_/M and S phases of the cell cycle suggestive of cell cycle inhibition. The nuclei of all the AgNP treated cells showed a dose-dependent increase in nanoparticles following non-ionic detergent treatment in which the nuclei retained extra-nuclear AgNP, suggesting that nanoparticles were attached to the nuclei or cytoplasm and not removed by detergent lysis. In summary, positively charged AgNP-bPEI increased particle cellular uptake. Particles agglomerated in the peri-nuclear region, increased mitochondrial toxicity, disturbed the cell cycle, and caused abnormal adherence of extranuclear material to the nucleus after detergent lysis of cells. These results illustrate the importance of nanoparticle surface coatings and charge in determining potentially toxic cellular interactions.

## Introduction

Engineered nanomaterials are increasingly used in industry and commerce for a wide range of potentially beneficial and profitable applications. Commercial nanoparticles NP have been designed for use in specific applications by varying their particle composition, size and coatings. The applications of nanoparticles in products, and the particle properties, influence the potential for release of particles from products and, in turn, the potential for inadvertent exposures and toxic reactions [1]. The size and composition of nanoparticles are important factors controlling their uptake into cells and potential for toxicity [2–10]. Because the primary interface between a nanoparticle and a cell occurs at the surface of the nanomaterial, one of the most influential features of nanoparticle bio-distribution and toxicity may be the particle surface coatings. Among other things, the particle surface coatings control surface charge, hydrophilic or hydrophobic nature, reactivity, agglomeration, dispersion stability in suspension media and sedimentation [11–18]. These factors ultimately will determine the potential toxicity of a particle.

The ability to study cellular uptake and distribution of nanoparticles requires the technological capability to detect the location of nanoparticles in the cell and to quantify cellular nanoparticle uptake. Previously, using darkfield microscopy we observed that silver and titanium dioxide nanoparticles readily accumulated with cells in tissue culture [19–25]. To enhance the detection of small nanoparticles we illuminated nanoparticles with a UV and blue wavelength rich Xenon light source in darkfield illumination. Because scatter intensity varies with the inverse 4^th^ power of the wavelength, shorter wavelength illumination will reveal smaller nanoparticles than red rich halogen illumination. By using a Xenon light source objects as small as 20 nm have been detected [21,26]. It was observed under darkfield imaging that TiO_2_ or Ag nanoparticles entered the cytoplasm, formed peri-nuclear agglomerations that grew as the exposure concentration increased, and eventually relocated in the lysosomes [18,27].

In addition to observing the location of nanoparticles within cells, it also is important to quantify their uptake. There was a relatively linear relationship between the dose of particles applied to cells and the uptake of particles measured by flow cytometry [22,23]. The relationship between nanoparticle dose and flow cytometry side scatter (SSC) suggests that it was an adequate descriptor of the number of nanoparticles absorbed by cells in culture [28–34].

The present studies evaluated the role of AgNP surface coatings on the nanoparticle’s cellular uptake, cellular disposition, and the interactions with cellular constituents. Darkfield microscopy was combined with fluorescent imaging of subcellular organelles to reveal the uptake and disposition/distribution of nanoparticles within the cell. Hyperspectral imaging provided a measure of the configuration of these particles in the cell. Specifically, we studied 80 nm AgNP stabilized with one of four coatings: citrate (CIT), branched polyethyleneimine (bPEI), polyvinylpyrrolidone (PVP), and polyethylene glycol (PEG)(S1 Table).

These particles have different surface charges with unique surfaces. In addition, one 80 nm gold nanoparticle coated with PVP was also evaluated as a comparison to AgNP-PVP. A culture of human-derived retinal pigment epithelial cells APRE-19 was used in these studies [19–23].

It was found that the incubation of both AgNP and AuNP with ARPE-19 cells resulted in a dose-dependent increase in the number of intracellular particles that could be observed by darkfield microscopy and flow cytometry. The flow cytometry intensity in the red fluorescence channels increased after 24-hour incubation with silver nanoparticles. The hyperspectral imaging of NP showed that intracellular nanoparticles had an increased wavelength of light reflected from them compared to extracellular nanoparticles. This observation in conjunction with a change in light scattering properties detected by hyperspectral imaging suggested that there was a change in the cellular nanoparticles’ agglomeration. This NP agglomeration change was accompanied by an increase in SPR [23, 35–37].

## Materials and methods

### Cell culture

Human-derived retinal pigment epithelial cells ARPE-19 cells ATCC, Manassas, Virginia were grown in T75 culture flasks in a 1:1 mixture of Dulbecco’s Modified Eagle’s Medium and Ham’s F-12 Nutrient Mixture DMEM/F-12 with 10% fetal bovine serum FBS. After reaching confluence, cells were trypsinized 0.05% trypsin, EDTA 0.02%, Sigma, and plated on chambered glass tissue culture slides 1 ml cell suspension per chamber, 2×10^5^ cells/ml. Cells were incubated for 24 h 37°C, 5% CO_2_ after plating, and then treated with nanoparticles.

### Nanoparticles

Four different coatings of 80 nm silver nanoparticles (AgNP) were compared in these studies: Branched Polyethyleneimine (AgNP-bPEI), citrate, (AgNP-CIT), Polyvinylpyrrolidone AgNP-PVP, and Polyethylene Glycol AgNP-PEG. and 80 nm Gold (AuNP-PVP) was also used as a metallic nanoparticle to compare with the 4 silver nanoparticles. Different suspensions of AgNP were obtained from nanoComposix (San Diego CA). Lot-specific physical/chemical characterization of each nanomaterial was provided by the supplier, including electron micrographic images and particle size distributions. The coated AgNP or the AuNP were obtained as a 1 mg/ml aqueous suspension. The NP were diluted in cell culture media containing FBS. ARPE-19 cells were incubated with the different types of AgNP at concentrations between 0.1 and 30 μg/ml for at 24 hr. prior to observation.

### Staining, fixation, and mounting

To identify structures and organelles within the cells, the cells were transfected with the following Invitrogen BacMan 2.0 reagents: Golgi GFP BacMan 2.0 (C10592), lysosome-RFP 2.0 (C10597), mitochondria-GFP 2.0 (C10600) or endoplasmic reticulum ER-GFP 2.0 (C10590) [Invitrogen, Eugene, Oregon]. Prior to fixation, the cells were counterstained with Cell Mask Orange Plasma stain (C10045, Invitrogen) to identify the cellular cytoplasmic area. The cells were washed and fixed with an equal amount of warm 4% paraformaldehyde (PF) in phosphate-buffered saline (PBS). The slides were then mounted with Prolong Gold, containing 10 μg/ml DAPI (P36935, Invitrogen) to stain the nuclei. After the mounting medium dried, the slides were sealed with Permount.

### Microscopy

A Nikon Ni upright microscope was used to observe cells in darkfield and fluorescence illumination. Fluorescence excitation cubes were used for DAPI, FITC, TRITC and Cyan GFP. The fifth cube holder space was intentionally left unoccupied to acquire a darkfield image without distortion from filters. The darkfield images were about 100 times brighter than the fluorescence images determined by their relative exposure times. The fluorescent and darkfield images were therefore taken sequentially at different exposure times and the images were combined using Nikon Elements software. Co-localization of the optical system was established with 0.5 μm Tetraspec beads and with 1 μm and 15 μm multi-wavelength fluorescent ring beads (Molecular Probes, Eugene Oregon). The xenon light supply was used with the darkfield images to obtain a bright light source that was optimized for the shorter wavelength excitation and better detection sensitivity. A GG 420 filter was put in the eyepieces to protect the user’s eyes from possible UV damage from the Xenon light source. [21–23].

A 60x Plan Fluor lens with an iris diaphragm to control the numerical aperture NA between 0.55 and 1.25 was used for imaging. Cellular details could be observed with this magnification while the background scatter was controlled by adjusting the diaphragm. The lower NA yielded good depth of field for bright nanoparticles, and the higher NA 1.25 yielded bright fluorescence images of cellular components. By balancing the fluorescence and darkfield signals, a sequential image could be acquired with the same NA setting approximately 0.8 NA. During this study, the darkfield images were obtained with a dry Plan Apo 20x lens (NA 0.75), a 20x multi-immersion (NA 0.75,) and 60x Plan Fluor with iris diaphragm (NA 1.25–0.55).

### PARISS hyperspectral imaging system overview

The Prism and Reflector Imaging Spectroscopy System (PARISS) (Lightform, Inc, Asheville, NC, USA) is a “push broom” hyperspectral imaging system that mounts on the video port of a microscope. The system is comprised of a prism-based imaging spectrograph, a CCD camera to record spectra, and a classic imaging camera to record a “visual” image of a field of view (FOV). All wavelengths in each spectrum are acquired and recorded simultaneously [38,39].

A computer-controlled microscope stage translates the sample on a slide, while a microscope objective projects an image of the FOV onto the slit of the imaging spectrograph. Light passes through the slit to the prism for spatially resolved spectral characterization. A typical translation of the FOV takes from a single to thousands of acquisitions, with around 300 being typical. The physical scan is only limited by the extent of objects of interest on the microscope slide. Following data processing, individual acquisitions are combined to form the final hyperspectral image.

Using reference materials, such as nanoparticle inside and outside cells, PARISS records the spectra of all objects that scatter light. The recorded spectra of all objects were classified using either unsupervised or supervised Spectral Waveform Cross Correlation Analysis (SWCCA). Selected classes were put into a “Reference Spectral Library" (RSL). Ubiquitous classes of spectra such as background, or those appearing less than 1% of the time in the FOV, were either deleted or “unchecked” to remove them from the final hyperspectral image.

### Creating a hyperspectral image of coated nanoparticles

1) Prepared samples on slides were “push broom” scanned, and all spectra presented by light scattering objects saved to disk. 2) Using SWCCA in either supervised or unsupervised, mode all spectra were classified according to a Minimum Correlation Coefficient (MCC). Limits were set on the number of possible classes by user selection of the MCC. 3) Classified spectra were inserted into an RSL. 4) Each class of spectrum in the RSL was pseudo-colored with a unique color of the operators choosing. Following completion of step 4 the system was ready to characterize unknowns as a function of their correlation with RSL spectra. The MCC was set to provide optimum correlation sensitivity.

#### Characterizing cells containing nanoparticles

Cells treated with the four coating types of AgNP samples were incubated for 24 hours and then were fixed with paraformaldehyde. Light scattered by AgNP located on the surface of the slide, and those that entered the cell were targets to be characterized with the PARISS system. The microscope was configured with a high NA oil condenser to maximize the photon density and increase the signal intensity. The objective lenses were either a 20x Plan Apo multi-immersion lens NA = 0.75, or a 60x Plan Fluor with a diaphragm iris collar NA 0.5–1.25 to adjust light for darkfield applications. A 75-watt Xenon light was used to illuminate the nanoparticles and act as the scattering light source. Neutral density filters were used to control brightness of the illuminant.

After slide preparation, PARISS physically scanned the slide and stored the spectra presented by all objects across the area of the scan. These spectra were correlated at a user selected MCC with spectra in the RSL. All spectra met the MCC were “painted” with the color of the correlated RSL spectrum onto a hyperspectral image. Spectra that failed to meet the MCC appeared in the hyperspectral image in gray. Clicking on a gray object in the hyperspectral image revealed its spectrum and the option of adding it to the RSL (S1 Fig).

### Flow cytometry

A Stratedigm S1000 flow cytometer (San Jose, California) containing 4 lasers 405 nm, 488 nm, 550nm, and 640nm was used. The flow cytometer was set up with a 488-nm excitation using a 50 mw 488 nm laser for scatter and fluorescence measurements and a 405nm 100 mw laser was used for cell cycle DNA measurements. The detection angles for the forward scatter FSC were between 1–8 degrees measured with a pin diode, while the side scatter SSC signal was obtained using a 40x lens with numerical aperture NA of 1.2. This configuration for FSC and SSC used quartz square flow cells like other flow cytometer analyzers. The analyzer instrument electronics necessitated that the first trigger be either forward or side scatter signal derived from the 488-nm laser. The unit contained a new generation of hybrid signal processing technology with a high signal-to-noise ratio, and high data acquisition speed. The doublet discrimination detection electronics helped to remove particles that simultaneously entered the flow cell during analysis.

This Stratedigm flow cytometer analyzer was checked daily for fluidic alignment using either Thermo Scientific 3 μm alignment beads [Cyto-Cal Multifluor plus Violet Intensity Calibrator, FC3MV] or the manufacturer recommended 3 μm Stratedigm QA alignment beads. The system was tested by the manufacturers QA procedures for the following parameters: fluidic flow, laser power, coefficient of variation CV in all the PMTs and the system electronics. The CVs of the test beads were generally between 1 and 3% and did not change greatly between the two flow rates: slow 25μL/min and fast 60μL/min. Both the CVs and mean values of the beads serve as a reference for instrument functionality [40–43].

### Measurement of cells and nuclei

The cells were measured with the following parameters. FSC, SSC, and 6 fluorescent parameters. The SPR was measured using the sensitive PMT representing the 690/40 nm channel which is equivalent to the FL3 detector 630 LP on a BD FACSCaliber used previously [23].

The cell cycle assay used mixed non-ionic detergent [NP-40 or Ipgal, Sigma, St. Louis, Mo] with cells to lyse the cytoplasm to obtain nuclei. The nuclei were then stained with Propidium Iodide (PI) 20 μg/ml or DAPI 10 μg/ml. The cells were lysed in 0.5% NP-40 or 0.5% Ipgal in Dulbecco’s PBS without Ca/Mg at a dilution of 2 parts lysing buffer to 1-part cells. The sample was put on ice after 20 minutes and then measured on the flow cytometer within a few hours. [43–46]. In addition to measuring PI or DAPI intensity to generate the cell cycle data [G_1_, S and G_2_/M], the scatter of nuclei was simultaneously obtained after nanoparticle treatment and is compared to control nuclei as illustrated in S2 Fig. The data were quantified using FCS express 6.0 with Multicycle to generate DNA cell cycle populations using the Dean and Jett model [43–46]. The technique is illustrated in S2 Fig that shows a clean nucleus from a control cell and nanoparticles attached to a cell that was treated with nanoparticles. This technique is illustrated with microscopic images from a detergnt lysed nuclei in S3 Fig. The image shows the cell accumulating clumped nanoparticles in the cytoplasm and after treatment with NP40 the image shows the nanoparticles are closely bound or associated with the nuclei.

### Concentration of nanoparticles

The concentrations of nanoparticles in samples obtained from the supplier was determined both from the manufacturer’s specifications and then checked in the laboratory by a procedure developed to count concentrated nanoparticles in suspension on a flow cytometer [24]. According to the manufacturer there were approximately 10^12^ particles per ml in each sample. The laboratory determination of particle counts involved sequentially dilution of samples with a fixed concentration of 1μm polystyrene beads until a dilute concentration could be counted on a flow cytometer without coincidence. The polystyrene beads were added to the cultures at concentrations between 0.1 μg/ml and 30 μg/ml. The method could then yield the count of NP per ml from the concentrated sample, and thereby allowed our laboratory to confirm the manufacture’s specifications of NP concentrations.

All particle samples were diluted to concentrations that ensured yields between 1000 and 6000 counts/sec, as determined by the flow cytometer software event counter. This translated to a concentration in the range of 10^6^ and 10^7^ counts per ml. If the concentration of particles greatly exceeded 10^7^, the observed count rate was reduced by the electronic circuitry of the flow cytometer to eliminate doublet counting, that resulted in the instrument rejecting many of the events that were deemed doublets by the suppression electronic software. The result was an instrument that paradoxically yielded fewer counts/sec in a concentrated sample than in a diluted sample. With this approach, we were able to minimize co-incident counting and obtain an accurate particle concentration measurement.

These diluted 1μm beads were mixed with the unknown AgNP sample that resulted in approximately 1000 counts/sec of beads which served as a reference in determining the proper measurement conditions for Ag nanoparticles. A 1 μm PS bead [Thermo Scientific # 4010A] was diluted to concentration that would yield about 400–500 counts/sec without Ag and Au nanoparticles present. The concentration of the 1 μm bead suspension was determined by mixing it with 2 μm counting beads 5x10^6^ and then comparing the percentages of the two beads in the SSC intensity histograms. With the 1 μm beads, the concentration of the diluent for the metal nanoparticles used in the flow cytometer was approximately 2-5x10^6^ particles/ml [24]. This was necessary to ensure that we were adding the same number of AgNP to each cell culture. The side scatter intensity data is dependent on the AgNP dose added to each culture.

## Results

**Fig 1:**
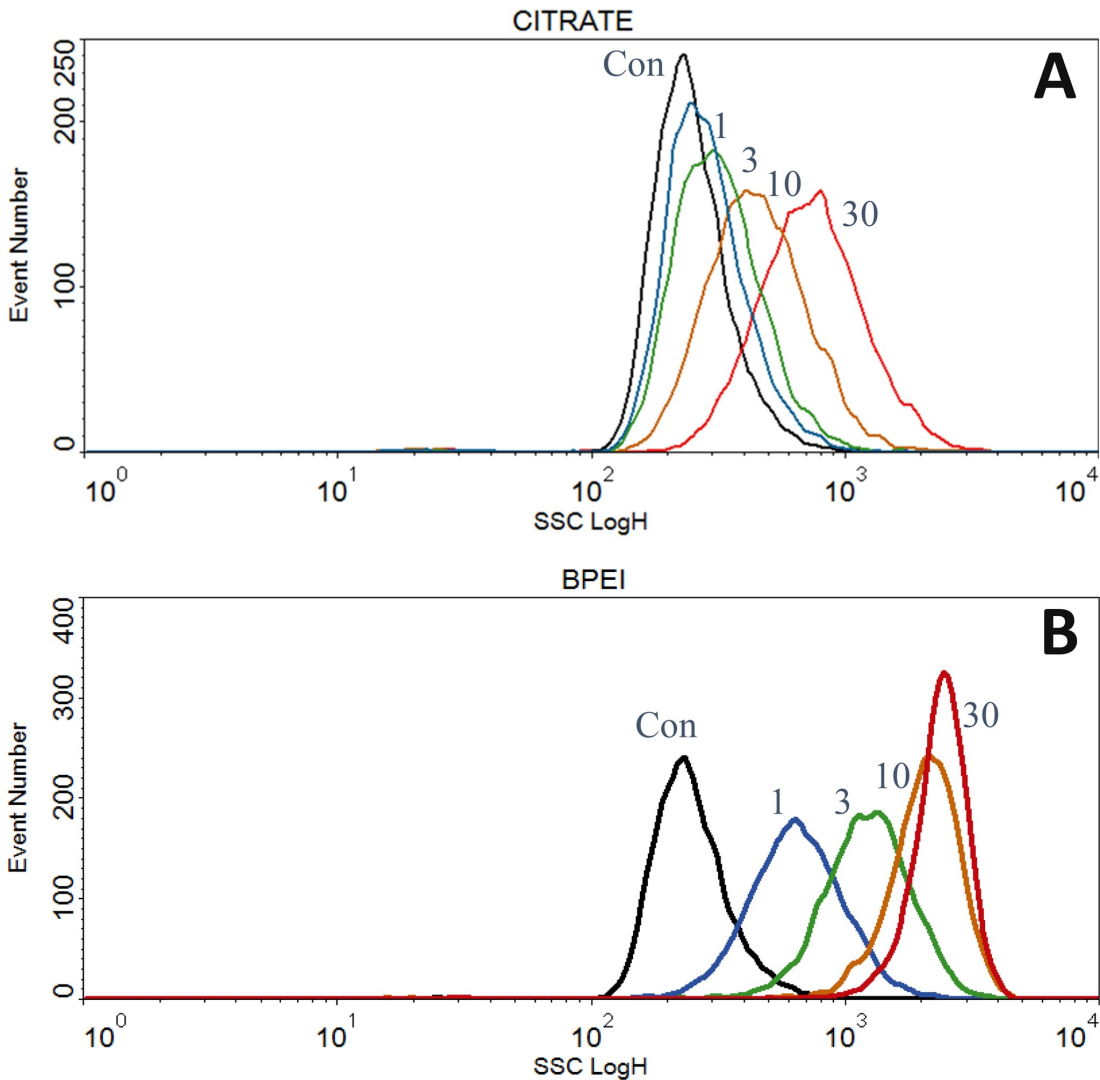
Flow cytometry side scatter ssc measurements of AgNP treated cells. Side scatter SSC measurement of cells were made after treatment with AgNP- CIT and AgNP-bPEI doses between 1μg/ml and 30 μg/ml for 24 hours. AgNP-CIT (Fig 1A, top) and AgNP-bPEI (Fig 1B, bottom).

**Fig 2:**
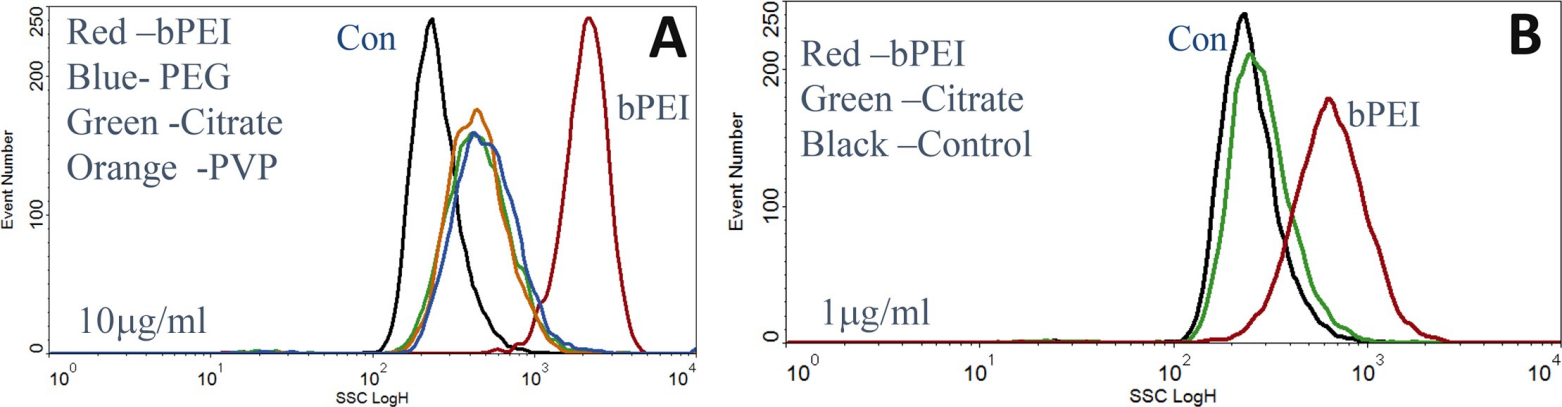
SSC measurements of AgNP-bPEI treated cells compared to other AgNP treated cells. Comparison of SSC of the four AgNP compounds at 10 μg/ml A, left and 1 μg/ml B, right. The AgNP-bPEI showed higher values suggesting greater accumulated than the other compounds at 1μg/ml and 10 μg/ml.

Four types of Ag nanoparticles with different surface coatings AgNP-bPEI, AgNP-CIT AgNP-PEG and AgNP-PVP having unique properties were added at concentrations between 1 μg/ml and 30 μg/ml to adherent ARPE-19 retinal epithelial cells for 24 hours (S1 Table). Au-PVP, a metallic particle like silver metallic nanoparticles was also measured as a comparison to the 4 silver nanoparticles. After 24 hours, side scatter (SSC), forward scatter (FSC) and far red fluorescence were measured on suspension cells using a flow cytometer. There was a dose dependent increase in SSC for the four AgNP between 1 and 30 μg/ml doses (Figs 1 and 2 and Table 1). Fig 1 shows the dose dependent uptake of AgNP-CIT (Fig 1A) and AgNP-bPEI (Fig 1B).

**Table 1:** SSC measurements of cells treated with AgNP and AuNP.

Dose μg	bPEI	Citrate	PVP	PEG	AU
**1**	**5.2**	**2.3**	**ND**	**ND**	**ND**
**3**	**9.5**	**2.6**	**1.5**	**1.4**	**1.4**
**10**	**15.5**	**3.7**	**2.0**	**1.8**	**1.9**
**30**	**17.5**	**6.0**	**3.1**	**2.4**	**2.5**

### SSC vs. dose

Cells were treated with four types of AgNP and AuNP-PVP. The AgNP-bPEI had about 3-6-fold more SSC than the other compounds. This observation was presumed to be related to the cellular accumulation of AgNP.

Cells treated with AgNP-bPEI scatter more light at all doses than do cells treated with AgNP-CIT. Cells treated with AgNP-PEG and AgNP-PVP were similar in their SSC dose response to AgNP-CIT. The forward scatter decreased slightly with each of the four compounds, as was previously reported with TiO_2_ and AgNP-PVP [22,23].

The relative SSC data comparing the four AgNP compounds and AuNP-PVP are shown in Table 1. At all the NP dose levels tested, there was an increase in SSC of the AgNP treated cells compared to control cells.

A comparison of the four types of AgNP are shown using doses of 10 μg/ml in Fig 2A and 1 μg/ml in Fig 2B. The AgNP-bPEI had more SSC than the other coatings at all comparable doses tested (Figs 1 and 2 and Table 1). The SSC derived from AgNP-bPEI was about 3–6 times higher than the other AgNP compounds and about 15 times greater than control cells at 10 μg/ml (Table 1, Fig 1A). Gold nanoparticles (AuNP-PVP) scattered light like AgNP-PVP and AgNP-PEG at equivalent doses.

If the concentration of AgNP-bPEI obtained from the supplier (nanoComposix) was much greater than the other AgNP compounds, it would explain the observed increased SSC with AgNP-bPEI. However, it was found that the concentrations of particles (1 mg/ml) determined by flow cytometry was within the range reported by the manufacturer. The AgNP-bPEI was even about 10% less in concentration than the other AgNP coated particles.

**Fig 3:**
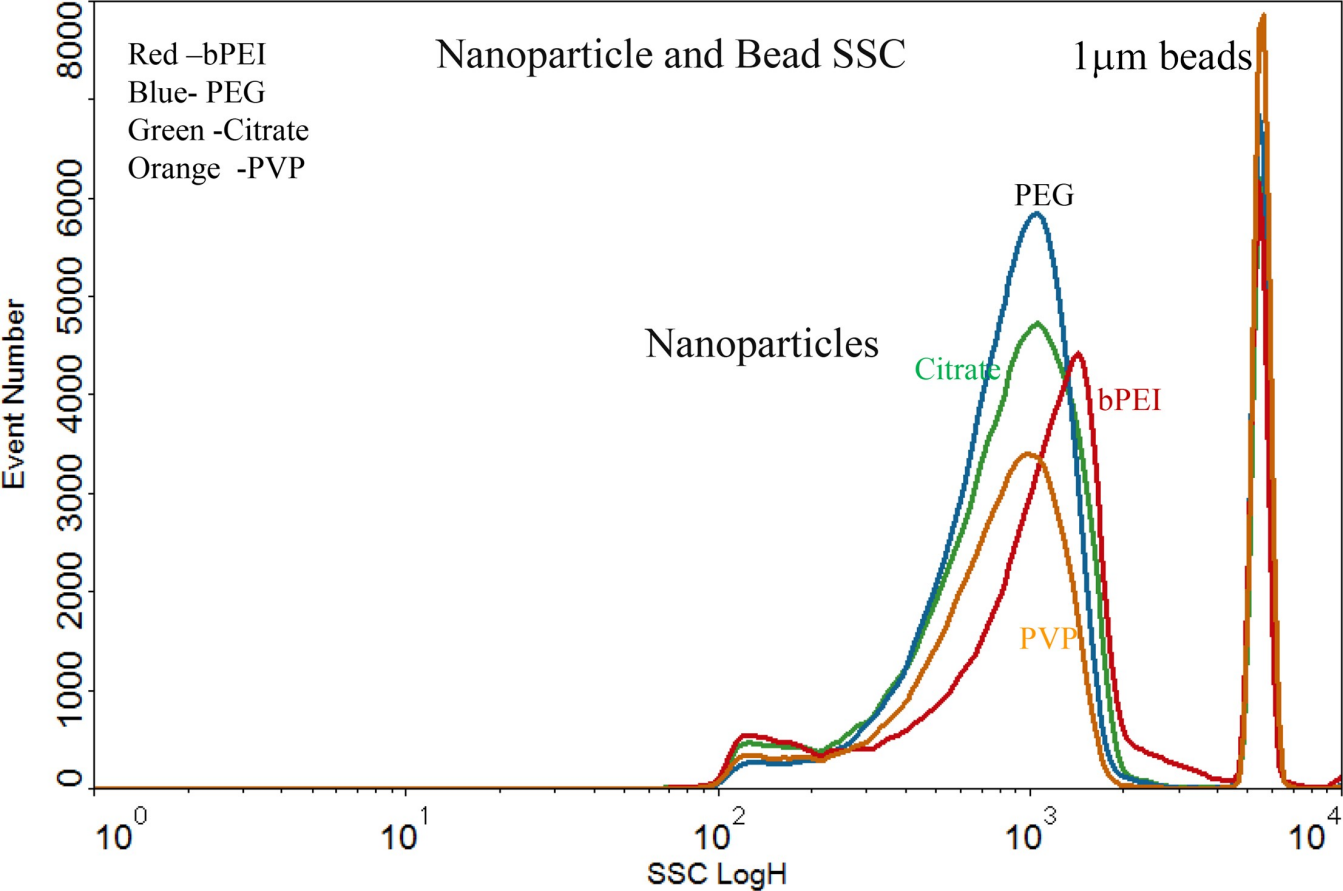
Comparison of nanoparticle SSC intensity of 4 AgNP and 1μm beads. Side scatter comparison of 4 types of 80 nm AgNP particles measured simultaneously with 1μm micron polystyrene beads. The AgNP-bPEI particles had slightly more SSC than the other 3 types of particles. These data were derived by diluting the AgNP with a defined concentration of beads until the AgNP beads were successively diluted to 10^6^ counts per ml.

The size of the different 80 nm AgNP in suspension were similar (Fig 3). The 1μm counting beads was measured simultaneous with the AgNP to insure the concentration of the particles were in a measurable range and not generating artifacts from coincidence counting or “Swarming” [47].

In addition to the side scatter measurements, emissions in the far-red channel (690/40 nm) were simultaneously measured using a red sensitive photomultiplier tube (PMT). The far-red fluorescent signal was designated as surface plasmonic resonance (SPR). The AgNP-CIT and AgNP-bPEI displayed greater SPR than the AgNP-PVP or AgNP-PEG. At the 10 μg/ml dose, the amount of SPR in the far-red channel (690/40nm, range 670–710 nm) was approximately 18 times greater for AgNP-CIT while AgNP-bPEI was about 10 times greater than control cells. Cells treated with PVP and PEG had about 2–3 times greater intensity than control cells treated at a dose of 10 μg/ml. Gold coated NP showed no increase in far red fluorescence, resembling control cells SPR fluorescence intensity (Table 2).

**Table 2:** Surface Plasmonic Resonance (SPR) vs dose.

Dose μg/ml	BEPI	Citrate	PVP	PEG	AU-PVP
**1**	**2.4**	**1.9**	**ND**	**ND**	**ND**
**3**	**3.5**	**2.8**	**1.1**	**1.2**	**1.1**
**10**	**9.5**	**17.8**	**3.5**	**2.8**	**0.9**
**30**	**55.5**	**104.6**	**16.3**	**25.0**	**.09**

## Surface Plasmonic Resonance vs. dose

Surface Plasmonic Resonance (SPR) vs. dose of the 4 AgNP compounds and AuNP-PVP at doses between 1 μg/ml and 30 μg/ml. The Ag-CIT had the most SPR followed by AgNP-bPEI, AgNP-PEG, and AgNP-PVP. AuNP-PVP showed no SPR. The AgNP-CIT treated cells were about 100-fold greater than control cells while the AgNP-bPEI treated cells were about 55-fold greater than control cells at the highest dose used 30 μg/ml.

**Fig 4:**
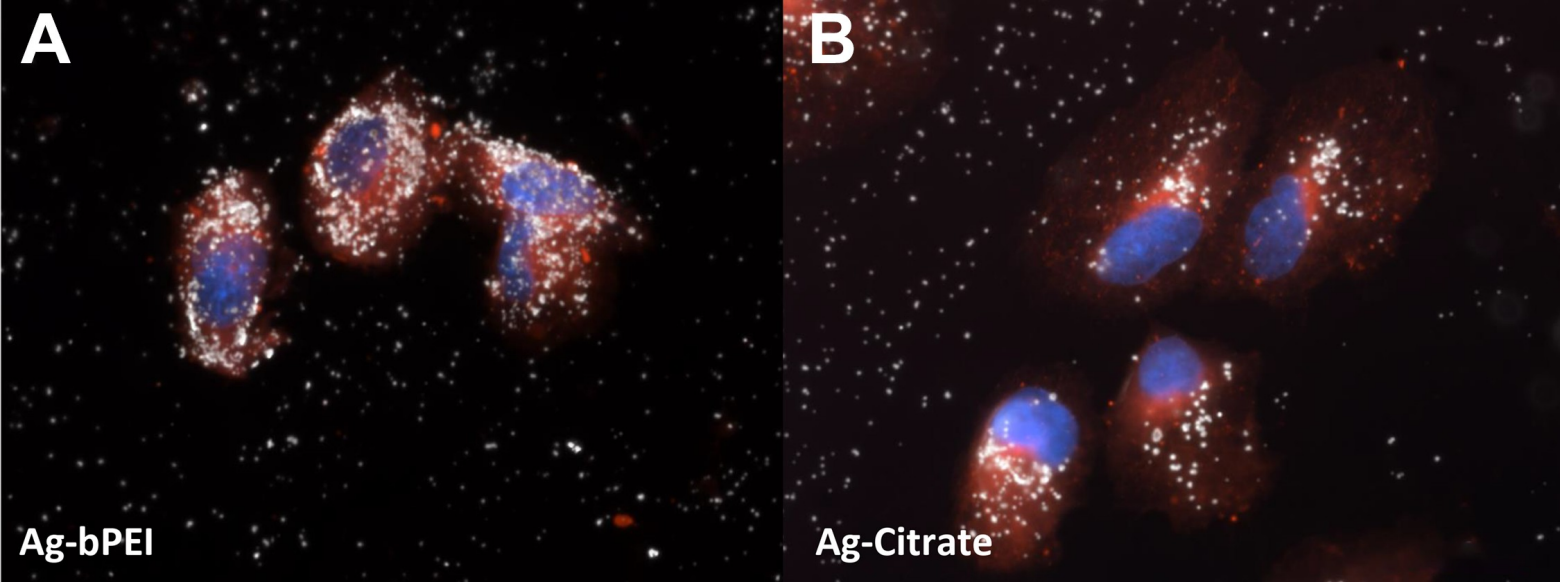
Microscopic observation of bPEI and citrate coated NP. Microscopic images of cells incubated with AgNP-bPEI (Fig 4A) and AgNP-CIT (Fig 4B) for 24 hours. The nuclei were stained with DAPI and the cytoplasm was stained with cell mask orange. The AgNP are represented by the white spots in the darkfield microscopy images. es. Magnification 200x.

Microscopic observation of the AgNP inside cells using darkfield microscopy revealed the relative uptake of NP. Fig 4 shows that there was a greater uptake of 3 μg/ml nanoparticles in the AgNP-bPEI treated cells compared to 3 μg/ml AgNP-CIT treated cells. The other AgNP compounds showed similar decreased uptake compared to AgNP-bPEI treated cells (Fig 4).

**Fig 5:**
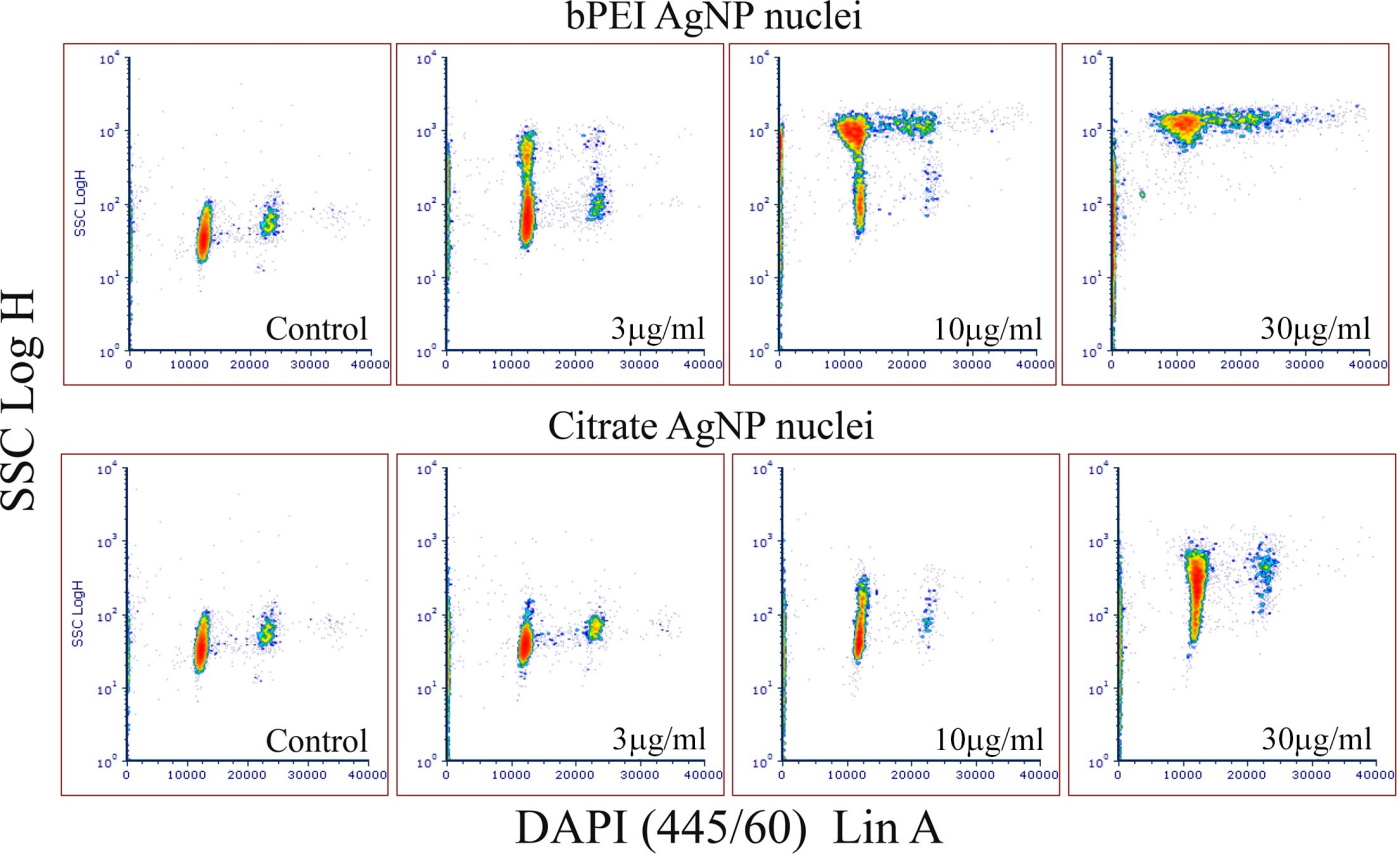
Flow cytometry cytograms of nuclei treated with AgNP-bPEI and AgNP-CIT. Flow cytometry comparison of DAPI stained nuclei derived from cells treated with AgNP-bPEI or AgNP-CIT at doses between 1μg/ml and 30 μg/ml. Each successive higher dose resulted in greater amount of side scatter. This was presumably due to higher uptake of AgNP as shown in the cell scatter data (Figs 1 and 2) and the lack of complete cell lysis at all doses.

**Fig 6:**
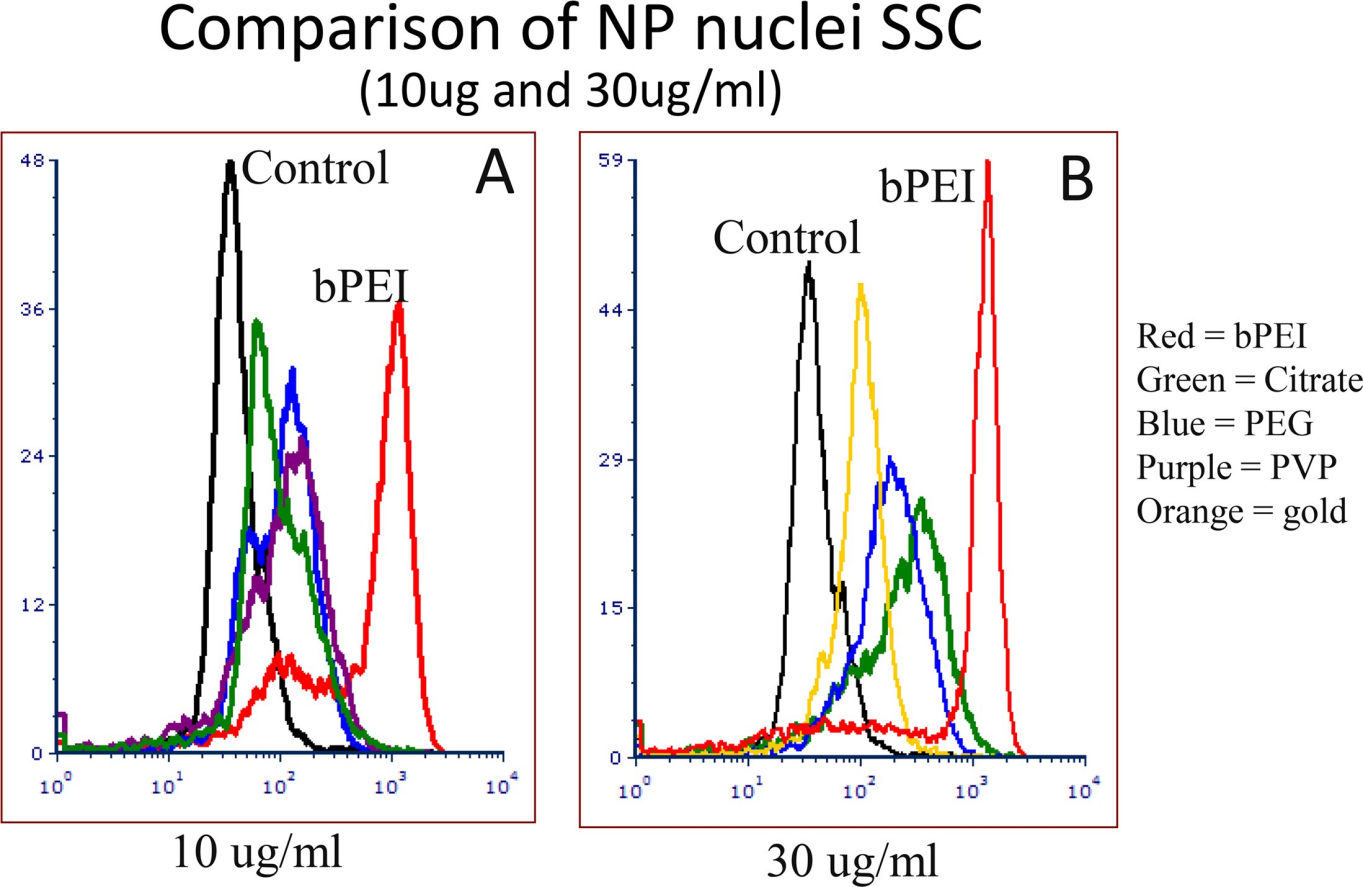
Comparison of nuclei SSC histograms from NP treated cells. Histograms of nuclei derived from cells treated with the 4 types of AgNP at 10 μg/ml (Fig 6A) and 30 μg/ml (Fig 6B). All samples demonstrated an increase of scatter after AgNP incubation for 24 hours at doses between 1 and 30 μg/ml. All the AgNP showed greater SSC than the AuNP.

Non-ionic detergent (NP-40 or Ipgal) was incubated with cells yielding nuclei that were then stained with PI or DAPI generating the cell cycle data (G_1_, S and G_2_/M) using the flow cytometer. The percentages of each phase in the cell cycle was generated using the Dean and Jett model from the Multicycle program contained in the FCS Express 6 software [43]. In addition to measuring DNA intensity with DAPI (10μg/ml) staining, the side scatter of nuclei was simultaneous obtained from the 488 nm laser. The data are displayed in both cytogram (Fig 5) and histogram (Fig 6) formats. Fig 5 shows a sequential increase in scatter as the dose increases from 3 μg/ml to 30 μg/ml for nuclei derived from cells treated with AgNP-bPEI and AgNP-CIT. The Coefficient of variation (CV) of the G_1_population increased showing that some nuclei had more scatter than others presumably due to having more nanoparticles attached to the nuclei. Fig 6 shows the relative scatter difference of nuclei derived from cells treated with different types of AgNP compounds. The AgNP-bPEI had greater scatter than the other AgNP compounds. These particles were not observed inside the nucleus of AgNP treated cells under darkfield microscopy suggesting that when the nuclei from treated cells are derived for cell cycle analysis they may have peri-nuclear cytoplasmic tabs containing nanoparticles, which ultimately increases the nuclear scatter.

S2 Fig and S3 Fig show the cells and detergent lysed nuclei with nanoparticles attached the nuclei. The cells have nanoparticles contained in the cytoplasm while the nuclei have the nanoparticles associated closely with the surface of the nuclei either through a direct bond or in cytoplasm remaining attached to nuclei after detergent non-ionic lysis.

The AgNP-bPEI showed the highest increased in nuclei side scatter which was related to the increased particle accumulation shown with cells (Figs 1 and 2 and Table 1). As the dose increased, the percentages of G_1_ for the bPEI treated cells decreased while the percentages for S and G_2_/M increased. (Table 3). The CV of the G_1_ increased as the dose increased indicating reduced accuracy in the DNA cell cycle modeling. It was not possible to acquire cell cycle distribution data from 30 μg/ml AgNP-bPEI. At higher concentrations, the Multicycle program could not generate accurate cell cycle phases due to broadening CV of the G_1_ population.

**Table 3:** Cell cycle phases of AgNP-bPEI.

Dose μg/ml	G1	S	G2/M	CV-G1
**0**	**83.9**	**4.7**	**11.4**	**3.25**
**1**	**79.7**	**8.0**	**12.4**	**3.67**
**3**	**81.9**	**5.8**	**12.3**	**3.66**
**10**	**70**	**16.2**	**13.8**	**8.70**
**30**	**60**	**ND**	**ND**	**14.7**

In contrast to AgNP-bPEI the cell cycle phases (G_1_ and S and G_2_/M) of AgNP-CIT, AgNP-PVP and AgNP-PEG did not vary greatly with the doses used. There was a slight increase in S and G_2_/M as the dose increased from 3μg/ml to 30 μg/ml. In summary it appears that the percentages of G_1_ decreased while the percentages of S and G_2_/M increased suggesting a retardation of the cell cycle progression with a block in the G_2_/M phases. The AuNP- PVP had less nuclei scatter than the other compounds.

## AgNP-bPEI cell cycle phases

The DNA histograms from AgNP-bPEI treated cells were used to calculate the percentages of cells in each phase of the cell cycle using the Multicycle program located in FCS express 6. The nuclei were derived by detergent lysis with NP40 and stained with DAPI (10μg/ml). There is a decrease in G_1_ and an increase in S and G_2_/M as the dose increased from 1 to 30μg/ml.

**Fig 7:**
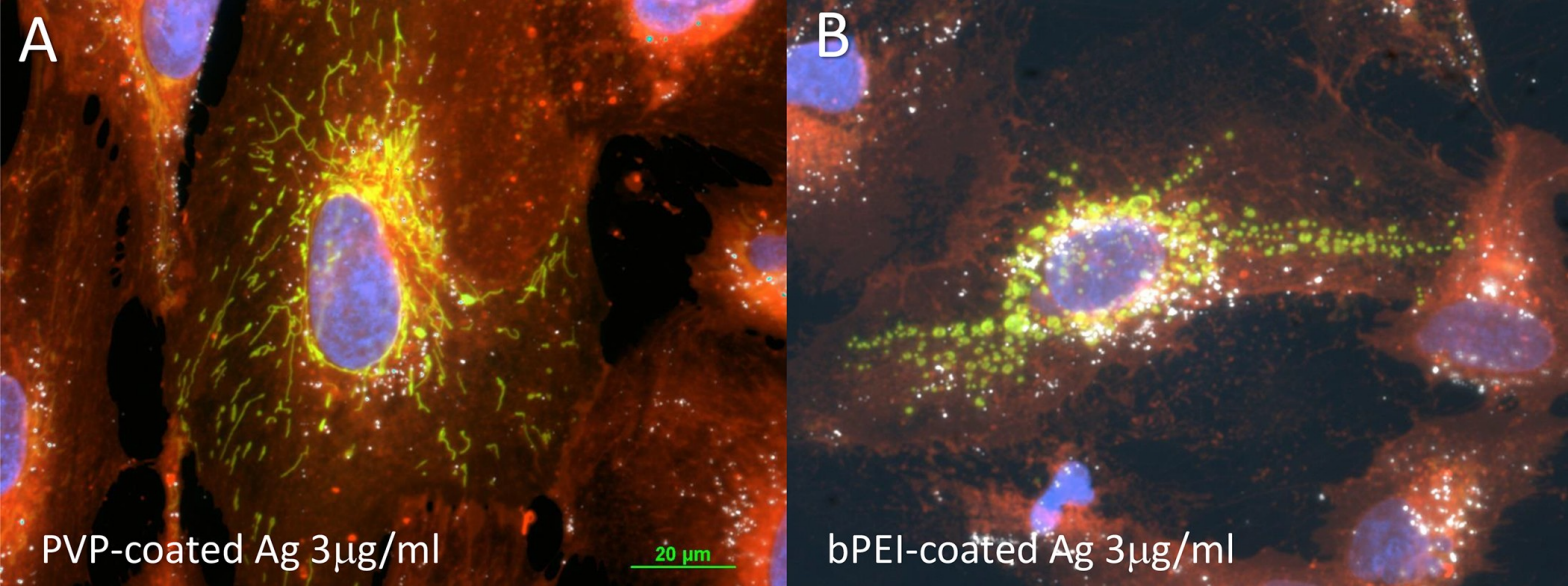
Mitochondria microscopy from AgNP-PVP and AgNP-bPEI treated cells. Comparison of the mitochondria shapes derived from cells treated with 3 μg/ml of AgNP-PVP (Fig 7A) and AgNP-bPEI (Fig 7B). The AgNP-bPEI had beaded shapes while mitochondria derived from the AgNP-PVP treated cells had a linear strand like formation of mitochondria. The cytoplasm is stained with cell mask orange (designated by red), the mitochondria transfected with GFP (designated by green) and the nuclei are stained with DAPI (designated by blue). The NP (designated by white) are accumulated throughout the cytoplasm and around the nuclei with the AgNP-bPEI samples. Magnification 600x.

The mitochondria-GFP staining was derived from transfecting the cells for 24 hours with BacMan 2.0 reagents (Thermo-Fisher, Invitrogen). The mitochondria shape was different in the AgNP-bPEI and the AgNP-citrate treated cells (Fig 7). The mitochondria in untreated cells and AgNP-CIT treated cells were thin strands that underwent fusion and fission (Fig 7A). However, after treatment with AgNP-bPEI the mitochondria changed shape and appeared as small beads (Fig 7B). It has been reported that AgNP-bPEI is toxic to cells at high concentrations, and this may be the reason for the observation that the mitochondria changed their shape [48].

**Fig 8:**
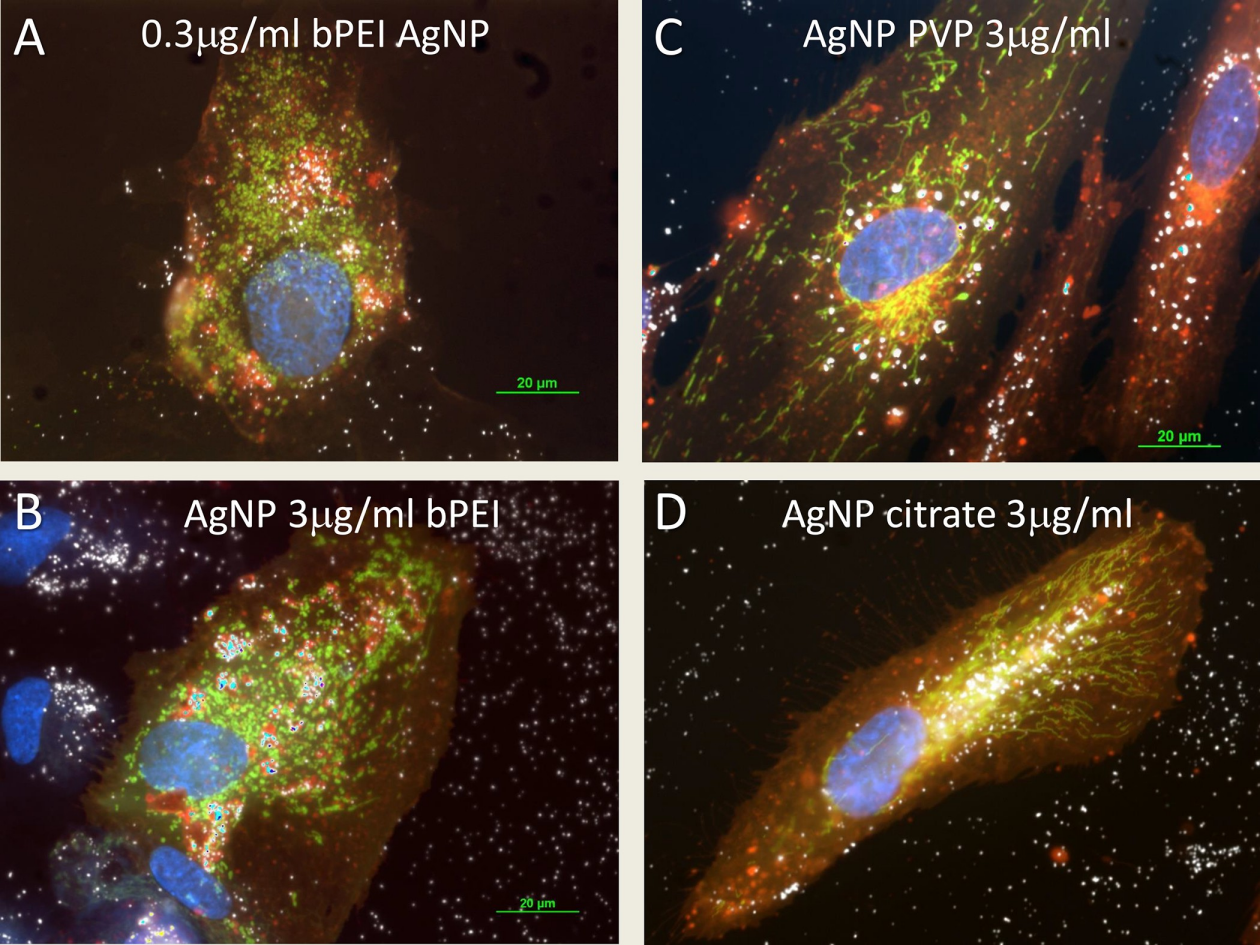
Microscopic comparison between different AgNP. Microscopic comparison of mitochondria after AgNP uptake into cells treated. with AgNP-bPEI, AgNP-PVP or AgNP-CIT for 24 hours. The AgNP-bPEI showed beaded structures of mitochondria at 0.3μg/ml (Fig 8A) and 3μg/ml (Fig 8B) while the AgNP-PVP 3μg/ml, (Fig 8C) and AgNP-CIT 3μg/ml,(Fig 8D) showed an elongated formation of mitochondria (designated by green color). The NP (designated by white color) are accumulated into the lysosomes (designated by red color) with the AgNP-bPEI samples while the NP are accumulated around the nuclei (Fig 8C) or in the ER (Fig 8D) with the other AgNP coated samples. The cytoplasm was stained with cell mask orange (red) and the nuclei are stained with DAPI (blue). Magnification 600x.

The cytoplasm was stained with cell mask orange and the nuclei stained with DAPI. Lysosomes were stained with RFP-BacMan probes. These microscopic images showed higher uptake of nanoparticles in the AgNP-bPEI compared to AgNP-CIT. The nanoparticles appear to be embedded in the region surrounding the nucleus and occupied by the endoplasmic reticulum (Figs 7 and 8). The cell mask orange plasma stain was shown to stain membranes which include both interior and exterior cellular membranes. An increased number of particles were observed with darkfield microscopy in Figs 4,7 and 8 as they reflect more light yielding a brighter and more intense signal. This was correlated to increased light scatter intensity measurements observed by flow cytometry with SSC illustrated in Figs 1 and 2.

### Hyperspectral imaging

**Fig 9:**
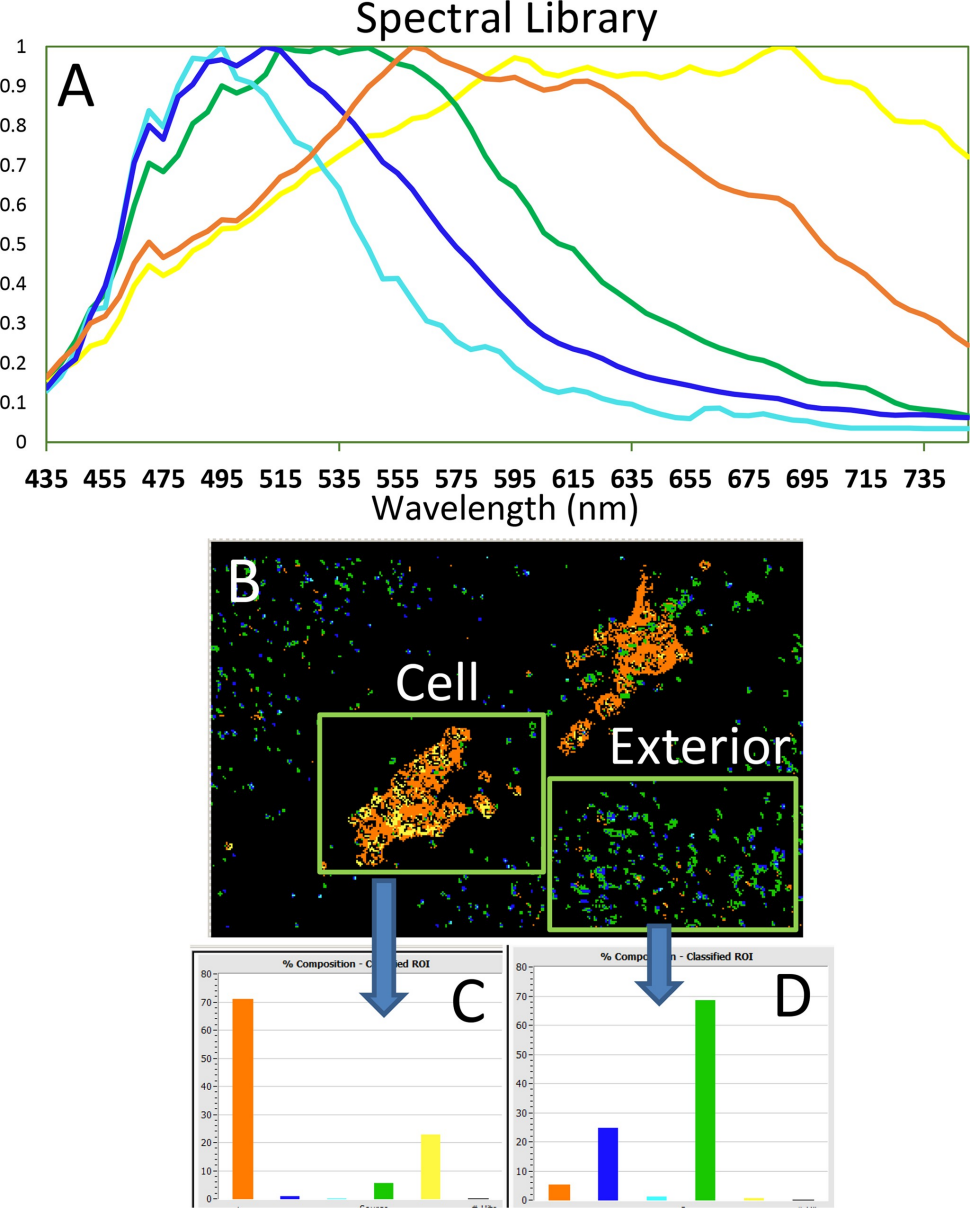
PARISS hyperspectral imaging of cell and exterior. The reference spectral library A was obtained by dividing the internal white xenon spectra with the spectra derived from individual NP or regions of NPs. A region of interest (ROI) was made around in the cell and another ROI was made on the exterior of the cell (Fig 9B) to determine the amount of reference spectra shown in Fig 9A that existed in these two regions that are expressed in a histogram format (Fig 9C and 9D).

The four different types of nanoparticles were investigated using the PARISS hyperspectral imaging system. To evaluate data derived from the PARISS hyperspectral imaging equipment the system was set up for darkfield illumination using a 75-watt xenon light source. A library of spectra was derived from light that was scattered by AgNP within the cell and from particles resting on the surface of the slide (Fig 9). The reflectance spectra had relatively shorter wavelengths designated by blue and green pseudo-colors outside of the cell while inside the cell the NP reflectance spectra yielded longer wavelengths designated by orange and yellow pseudo-colors.

A spectrum of the Xenon light source was obtained with no cells present on the slide and this spectrum was divided into the individual spectra of the library to obtain reflectance spectra library (Fig 9A). Observation of the nanoparticles on the slide surface and within the cells are shown in a representative image of AgNP-PVP treated cells. The spectra of AgNP observed outside the cell had shorter wavelengths than spectra derived from AgNP that entered the cell. The dark-field scatter image of a cell is pseudo-colored using the spectral library (Fig 9B). The histogram percentages of each spectra from the cell (Fig 9C) and the region outside of the cell on the slide surface are shown in histograms format (Fig 9D) as percentages of different pixels represented by the colors shown in Fig 9A. The region in the cell’s interior contains mostly longer wavelength spectra designated by orange and yellow colors, while the regions outside the cell on the slide surface is represented by blue and green spectra that peaked at shorter wavelengths.

**Fig 10:**
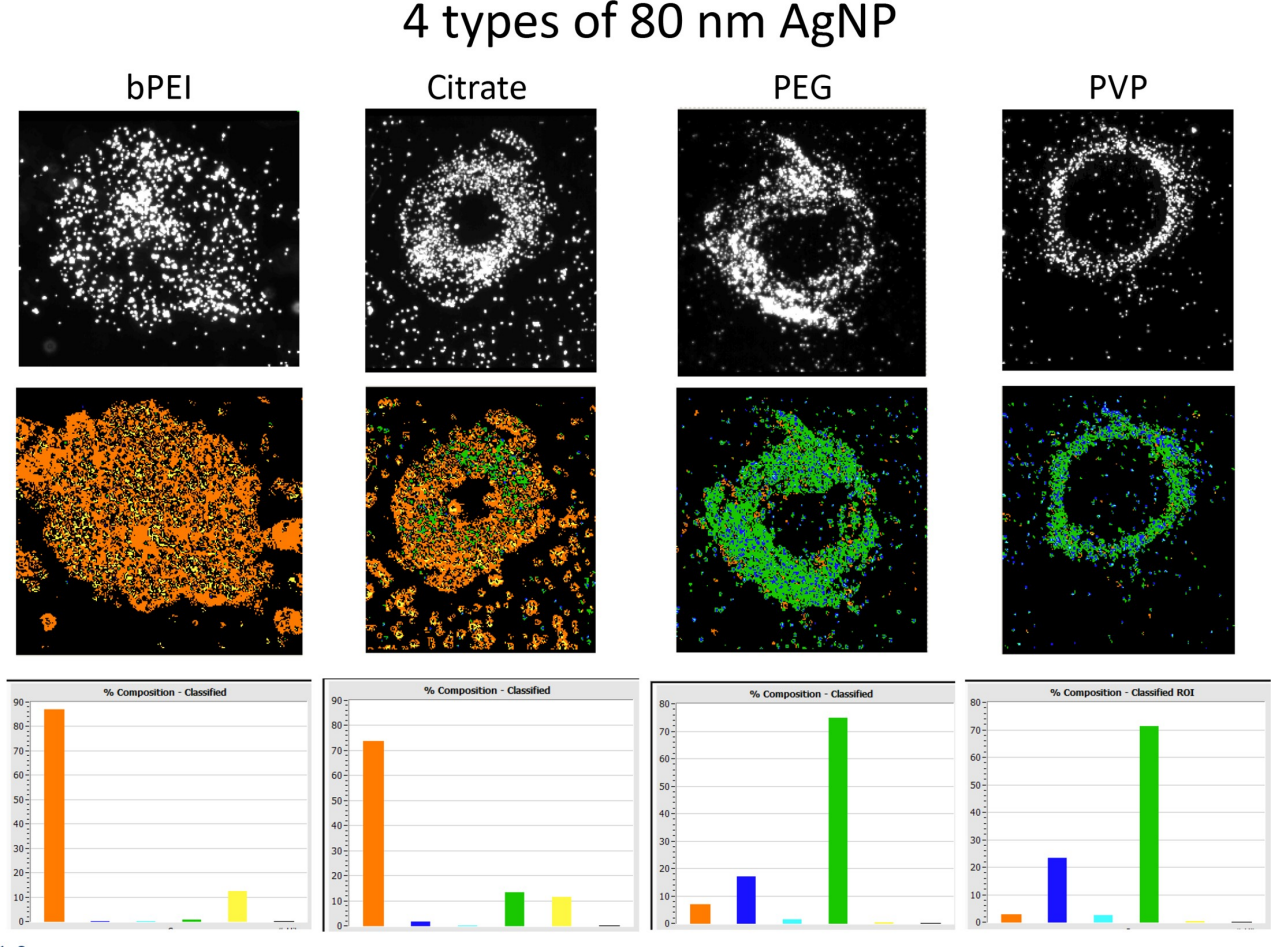
Hyperspectral image comparison of 4 types of AgNP. The top row in Fig 10 consists of scattered images from the PARISS hyperspectral system, while the middle row presents pseudo-colored images using the spectral library shown in Fig 9A. The percentage of each type of spectra is shown in the histograms displayed on the bottom row of Fig 10. The magnification of the images was 600x.

During these studies, many of the cells treated with AgNP had showed a circular structure consisting of concentrated NP on the slides that was not associated with cells (Fig 10). Circles consisting of nanoparticles NP were observed using darkfield illumination on the slides after the ARPE-19 cells were exposed to each of the four compounds at 3 μg/ml NP (top row). These NP circles were pseudo-colored with the spectral reference l library shown in Fig 9A and were represented in the middle row of Fig 10. The percentages of each reference spectrum appearing in these circular structures are shown in the corresponding histograms (bottom row). The AgNP-bPEI and the AgNP-CIT circles had relatively longer wavelengths represented by primarily orange and yellow pseudo-colored spectra while the AgNP-PVP and AgNP-PEG circles had relatively shorter wavelengths represented by primarily blue and green pseudo-colored spectra. This implies that the agglomeration of the particles in the AgNP circles was different with the AgNP-bPEI and AgNP-CIT compared to the AgNP-PVP and Ag-PEG. This agglomeration difference between the different AgNP indicated that the degree and type of agglomeration occurred which could affect the scattering wavelength.

Upon the evaluation of the reflectance of light from these circles, it was found that AgNP-PVP and AgNP-PEG had spectra that peaked in the shorter range while AgNP-CIT and AgNP-bPEI had spectra that tended to peak in the longer range. The nanoparticle circles derived from PVP, PEG, and citrate NP incubations with cells had a hollow core while the branched had a circle the consisted of particles that had equally distributed concentrations without a hollow core center. It is not clear what is generating these circular structures, but it appears from past research in our laboratory that it may be due to adherent proteins that selectively bind nanoparticles during the mitosis process. The positive charge AgNP-bPEI has a different pattern than the negatively or neutral charged particles and may be responsible for the different patterns that was observed.

## Discussion

The primary aim of this paper was to compare the cellular uptake and distribution of AgNP with different capping agents. Four types of 80 nm AgNP were used: Branched Polyethyleneimine (bPEI), citrate (CIT), Polyvinylpyrrolidone (PVP), and Polyethylene Glycol (PEG) (S1 Table). An 80 nm gold NP (AuNP-PVP) was compared to the other AgNP metallic particles. Measures of NP uptake included flow cytometry, fluorescence, dark field microscopic imaging and PARISS hyperspectral imaging.

The SSC signal was influenced by the type of coating agents i.e. the positively charged AgNP-bPEI accumulated in cells to greater extent compared to the other AgNP studied. All coating types of AgNP entered the cells and were detected by a dose dependent increase in the SSC intensity. The AgNP-CIT and AgNP-bPEI agglomerates, both showed increased emissions in far-red wavelengths indicative of a high surface plasmonic resonance. Silver nanoparticles that settled on the surface of the slides could be observed with darkfield microscopy. These NP on the slide surface reflected relatively shorter wavelengths of light indicating the presence of individual particles or small agglomerates of AgNP. In contrast the silver nanoparticles that entered the cells usually formed larger particles by agglomeration that reflected longer wavelengths of light.

In summary, our research studied the AgNP using the light scatter parameter of the flow cytometer as a measure of cellular uptake [22,23]. The positive charged AgNP-bPEI was accumulated more readily into cells than the other AgNP studied (Figs 1 and 2, Table 1). AgNP-bPEI was associated with changes in mitochondrial shape, high level of nanoparticles binding to the nucleus after non-ionic cell lysis, and interruption of normal cell cycling. Correlated with the AgNP uptake, was the detection of SPR changes using flow cytometry. The degree of agglomeration appeared to affect the SPR intensity detected by flow cytometry with AgNP-bPEI and AgNP-CIT being much greater than AgNP-PEG and AgNP-PVP. Since the doses of nanoparticles used were relatively low, (usually between 1–10μg/ml), there was only limited cytotoxicity observed in 24 hours of cell culture. However, using a fluorescence microscopy, mitochondria transfected with GFP were observed to be converted from long strands into small round balls which is suggestive of toxicity and possibly a loss of function. Additional toxicity of AgNP-bPEI was demonstrated as a decrease in G_1_ phase and increase in G_2_/M and S phases over a 24-hour period, suggesting an inhibition of the normal cell cycle progression. Thus, the effects of AgNP in a cell culture system could be readily observed and measured, enabling a comparative analysis of the influence of different types of nanoparticle coating materials. A major conclusion from these studies was that the positively charged AgNP-bPEI were accumulated more readily into cells and were generally more toxic to cells than AgNP with other surface coatings.

The different capping agents of Ag nanoparticles contained unique surfaces that were designed to help increase their stability in suspensions and enhance their use in specific applications [13, 14, 17]. For example, PEG coatings deter immunological identification of nanoform drugs and thereby increase their biological half-lives. PVP coatings generally increase stability of nanoparticle suspensions. Positively charged or negatively charged coatings (bPEI and citrate, respectively) provide electrostatic advantages to attach oppositely charged molecules to the nanoparticles, depending on the specific application desired (S1 Table). Thus, biomedical and commercial applications of nanoparticles could involve a large variety of coatings, which profoundly influence the behavior of particles and their potential toxicity. Our data suggests positively charged coatings will be associated with higher potential of accumulating AgNP in cells, which should be a consideration for the AgNP production.

### Flow cytometry data

Previously, our laboratory demonstrated that ARPE-19 cells incubated with either TiO_2_ or AgNP absorbed particles inside the cells, which was detected as an increase of flow cytometer light side scatter (22, 23). The research in this manuscript compared AgNP with four types of capping agents that provided different surface charges and surface features. The uptake of AgNP-bPEI by the cell increased the side scatter of both whole cells (Figs 1 and 2) and isolated nuclei (Fig 6). This positively charged AgNP-bPEI had the greatest increase in side scatter, suggesting a high absorption of these particles. The negative and neutral compounds (AgNP-PEG, AgNP-PVP and AgNP-CIT) did not scatter as much light as AgNP-bPEI and were thus assumed not to be less absorbed by the cells compared to AgNP-bPEI.

There is an observation that NP attached to nuclei are increased in a does dependent manner after detergent lysis. One hypothesis to explain the association between the nanoparticles and nuclei is that the NP are imbedded in the ER and this bit of cytoplasm containing NP remains attached to the nuclei after non-ionic treatment. Another hypothesis that may explain the data is that the nuclei becomes extremely sticky binding free floating nanoparticles. There was an increased SSC intensity of the 80nm AgNP-bPEI particles measured on the flow cytometer (Fig 3). This may be due to the surface of the AgNP-bPEI which has rough features as designated by the branched name that suggests these NP will reflect more light than AgNP containing smoother surfaces of the Citrate, PEG and PVP. It is not clear if this roughness parameter effected the absorption or if the positive charge was the primary determinant for absorption.

### PARISS hyperspectral imaging

The optical properties of gold and silver nanoparticles are sensitive to particle size, shape, concentration, agglomeration state, and refractive index near the nanoparticle surface, which makes UV/VIS spectroscopy a valuable tool for identifying, characterizing, and studying nanomaterials. Other factors that might affect the wavelength spectra include the microscope configuration, light sources, objectives and mounting media. For many types of nanoparticles, the spectral wavelengths profiles can shift due to changes in the size, shape, coating, or agglomeration state. Blue-shifting refers to an electromagnetic response that is shifted towards shorter wavelengths (higher frequencies, higher energies) while red-shifting refers to shifts towards longer wavelengths. In our studies, AgNP-bPEI and AgNP-CIT appear to clump more inside the cells resulting in a red shift to longer wavelengths. By comparison AgNP-PVP and AgNP-PEG, which do not clump as readily in cells or solution, show a shift toward relatively shorter blue wavelengths. However, particle agglomeration will typically result in a spectral red shift. The surfaces of PVP and PEG are hard and not displaceable, while citrate and bPEI have surfaces which can be modified and exchanged with other molecules making them useful for specific biological applications. These surface features may be partially responsible for the biophysical differences that we observed with flow cytometry and PARISS imaging spectrophotometer instruments.

### Surface plasma resonance

The optical properties of silver nanoparticles change when the particles aggregate and the electrons near the surface become delocalized and are then shared among adjacent particles. When this occurs, the surface plasmon shifts to lower energies and the scattering peaks shifts to longer spectra. The SPR occurs when the conduction electrons on the nanoparticle surface undergo a collective oscillation after excitation at specific wavelengths of light [35,36]. This results in a strong absorption of light followed by oscillation of plasmonic nanoparticles and an increase in the scattering of light.

These SPR effects were observed to a greater amount with citrate and bPEI surface AgNP coatings compared with PVP and PEG surface coatings (Table 2). In our studies, AgNP-CIT demonstrated the most SPR fluorescence, followed by AgNP-bPEI, AgNP-PEG, and AgNP-PVP. Cells treated with AuNP-PVP coated showed no SPR in contrast to AgNP-PVP which did show some SPR in the far-red channel on a flow cytometer.

The PARISS hyperspectral imaging data on NP contained in the cells or on the slide showed that AgNP-CIT and AgNP-bPEI shifted the exciting light to longer wavelength spectra suggestive of higher agglomeration. In contrast, AgNP-PVP and AgNP-PEG shifted the light to relatively shorter wavelengths which was suggestive of less agglomeration indicated by the detection of less SPR intensity.

It appears that the PARISS hyperspectral imaging data correlates with the SPR flow cytometer data. The greater agglomeration that was observed with AgNP-bPEI and AgNP-CIT using the PARISS imaging equipment may be related to the flow cytometry data that showed an increased SPR and increased SSC intensity measurements. In summary, both techniques were influenced by the degree of particle agglomeration.

### Darkfield microscopy

Dark-field microscopy illumination describes a technique that can be used to enhance contrast in unstained samples. The incident beam is not captured directly by the objective lens, but the light that has been scattered by the sample is captured which results in a bright image with a dark background. Essentially, the scattered light that enters the objective lens will produce an image while the direct light entering the objective is blocked.

Darkfield microscopy has the potential to observe objects below the diffraction limit of the microscope (220 nm) and therefore is useful to observe nanoparticles that are less than 100 nm in size [22,25–27]. However, the major limitation of darkfield microscopy is that the sample has to be strongly illuminated. In the case of nanoparticles, blue biased light from xenon bulbs have higher energy and lower wavelengths making xenon superior to the red biased light derived from halogen bulbs. These halogen bulbs are contained in most microscopes as their primarily bright field light source. Conventional microscopy using differential interference microscopy (DIC) can only measure NP clumps which have physical sizes above the diffraction limits of the microscope (around 220 nm). Darkfield microscopy can observe 20 nm AgNP.

The most observable difference between the four types of AgNP by fluorescence microscopy was that AgNP-bPEI caused mitochondria to change from long strands in control cells or AgNP-PVP treated cells [Fig 7A] to a round shaped mitochondria in cells treated with AgNP-bPEI (Fig 7B). This fluorescence microscopy observation of mitochondrial shape changes may be related to the toxicity data that is present in the literature with AgNP-bPEI [49]. The amount of AgNP uptake by cells was also observed by darkfield microscopy (Figs 4, 7 and 8). There was a greater number of particles absorbed in cells l treated with AgNP-bPEI compared to cells treated with AgNP-CIT at the same 3μg/ml dose (Fig 4). Future expanded studies on mitochondria using TEM may shed more detailed information on the internal configuration of the mitochondria after interaction with positive and negative charged AgNP.

### Green chemistry

The coatings on silver particles described in this manuscript were made with chemical and physical synthesis by the supplier, nanoComposix. However, there is the possibility of stabilizing silver molecules by green synthesis with plant extracts and enzymes. These silver nanoparticles synthesized by plant extracts are economical and cost effective and conceivably provide a product that is better and safer and less toxic for humans and the environment. [50,51] The molecules have been reduced and stabilized by a combination of biomolecules. These surfaces on nanoparticles are ecofriendly and may be compatible with biological and pharmaceutical applications.

With these advantages if would be useful to compare the “green synthesized particles” with nanoComposix AgNP in regard to their toxicity, uptake, and flow cytometry light scatter and their effectiveness in biological applications. This comparison of green chemistry synthesized nanoparticles with nanoComposix produced silver nanoparticles would be a useful endeavor for our future research using the technology described in this manuscript.

## Conclusions

NP uptake, intercellular agglomeration, and morphological effects were monitored by microscopy, hyperspectral imaging, flow cytometry and surface plasmonic resonance. Flow cytometry SSC was again shown to be a good dosimeter of the relative uptake of metallic nanoparticles into cells with some coatings of AgNP being accumulated at a greater rate than other AgNP molecules. The flow cytometry measurements of side scatter of cells and nuclei showed that AgNP-bPEI was absorbed more than other compounds at all doses (Table 1 and Fig 1). The positive charged molecules were toxic to mitochondria. The agglomeration characteristics of the particles on the surface of slides and within the cells could be studied by the PARISS hyperspectral imaging system. These methods demonstrated an increase in SPR and a shift to longer wavelengths with increased agglomeration. In future studies, these methods employed in this manuscript may help in to understand how NP interact with cells. Future studies will continue to explore how the different AgNP surfaces effect the potential health risk due to their relative uptake and potential toxicity.

## Supporting information

S1 Fig PARISS imaging spectrophotometer work flow. An imaging spectrograph (PARISS) acquires the spectra presented by a field of view (FOV) as it is translated on a computer-controlled microscope stage. Spectral Waveform Cross Correlation Analysis (SWCCA) classifies all acquired spectra. A reference spectral library of the classified spectra is generated which is then used to classify and pseudo-color a hyperspectral image of the FOV.
(TIF)

S2 Fig Nuclei detergent lysis method for cell cycle analysis. The cells that were treated with AgNP show nanoparticles bound to the nucleus by microscopy and and increase in scatter by flow cytometry. The flow cytometry data using either PI or DAPI staining shows a dose dependent increase in side scatter in all phases of the cell cycle. The insert shows a cytogram representing the cell cycle stages with the black values being control and red values being the cellular sample that was treated with AgNP. The cell cycle of the nuclei was evaluated with the Multicycle program contained in the FCS express software (De Novo software, Los Angles, Ca).
(TIF)

S3 Fig Cells (A) were incubated with 10ug/ml TiO2 Degussa. The image on the left (B) shows the nuclei stained with DAPI surrounded by nanoparticles with dispersed nanoparticles in the cytoplasm. After detergent lysis the cytoplasm is largely removed and some of the nanoparticles are attached to the nuclei. Images were acquired sequentially with fluorescence derived from DAPI stained nuclei (blue) and nanoparticles (white) obtained from with darkfield illumination. The two images were combined using Nikon Elements 5.0. About 30 images of the round cells were taken with Nikon widefield imaging software allowing for the generation of a “Z” stack. The images were sharpened using an extended depth focusing algorithm.
(TIF)

S1 Table Characteristics of Ag coatings of nanoparticles. Derived from the nanoComposix web site https://nanocomposix.com.
(TIF)
