# Supplementary figures and images for: Biophysical comparison of four silver nanoparticles coatings using microscopy, hyperspectral imaging and flow cytometry
Source: PLoS One. 2019 Jul 31;14(7):e0219078. doi: 10.1371/journal.pone.0219078 (PMC6668787; doi:10.1371/journal.pone.0219078)

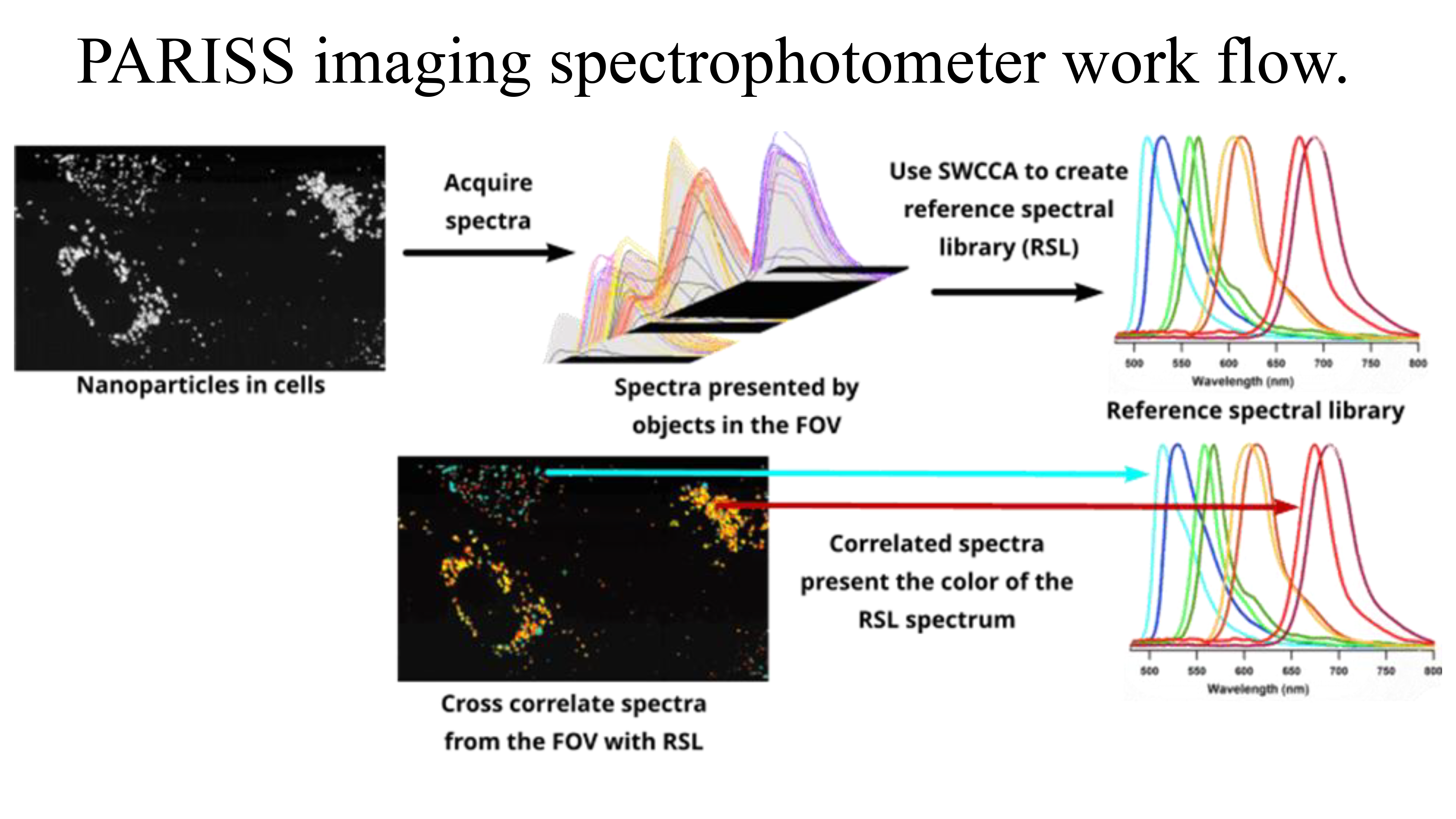

Supplement: S1 Fig — An imaging spectrograph (PARISS) acquires the spectra presented by a field of view (FOV) as it is translated on a computer-controlled microscope stage. Spectral Waveform Cross Correlation Analysis (SWCCA) classifies all acquired spectra. A reference spectral library of the classified spectra is generated which is then used to classify and pseudo-color a hyperspectral image of the FOV. (TIF) [file pone.0219078.s001.tif]

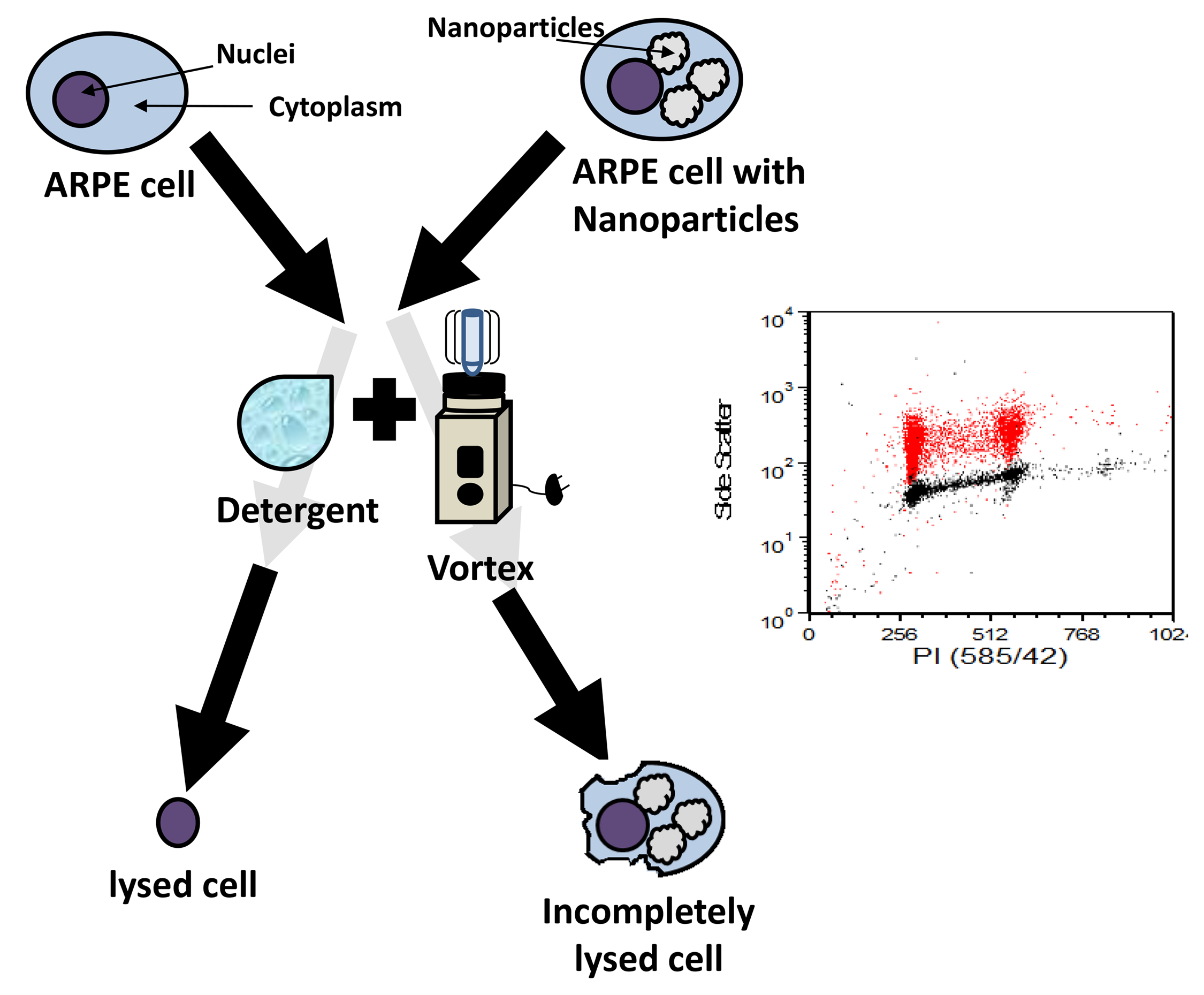

Supplement: S2 Fig — The cells that were treated with AgNP show nanoparticles bound to the nucleus by microscopy and and increase in scatter by flow cytometry. The flow cytometry data using either PI or DAPI staining shows a dose dependent increase in side scatter in all phases of the cell cycle. The insert shows a cytogram representing the cell cycle stages with the black values being control and red values being the cellular sample that was treated with AgNP. The cell cycle of the nuclei was evaluated with the Multicycle program contained in the FCS express software (De Novo software, Los Angles, Ca). (TIF) [file pone.0219078.s002.tif]

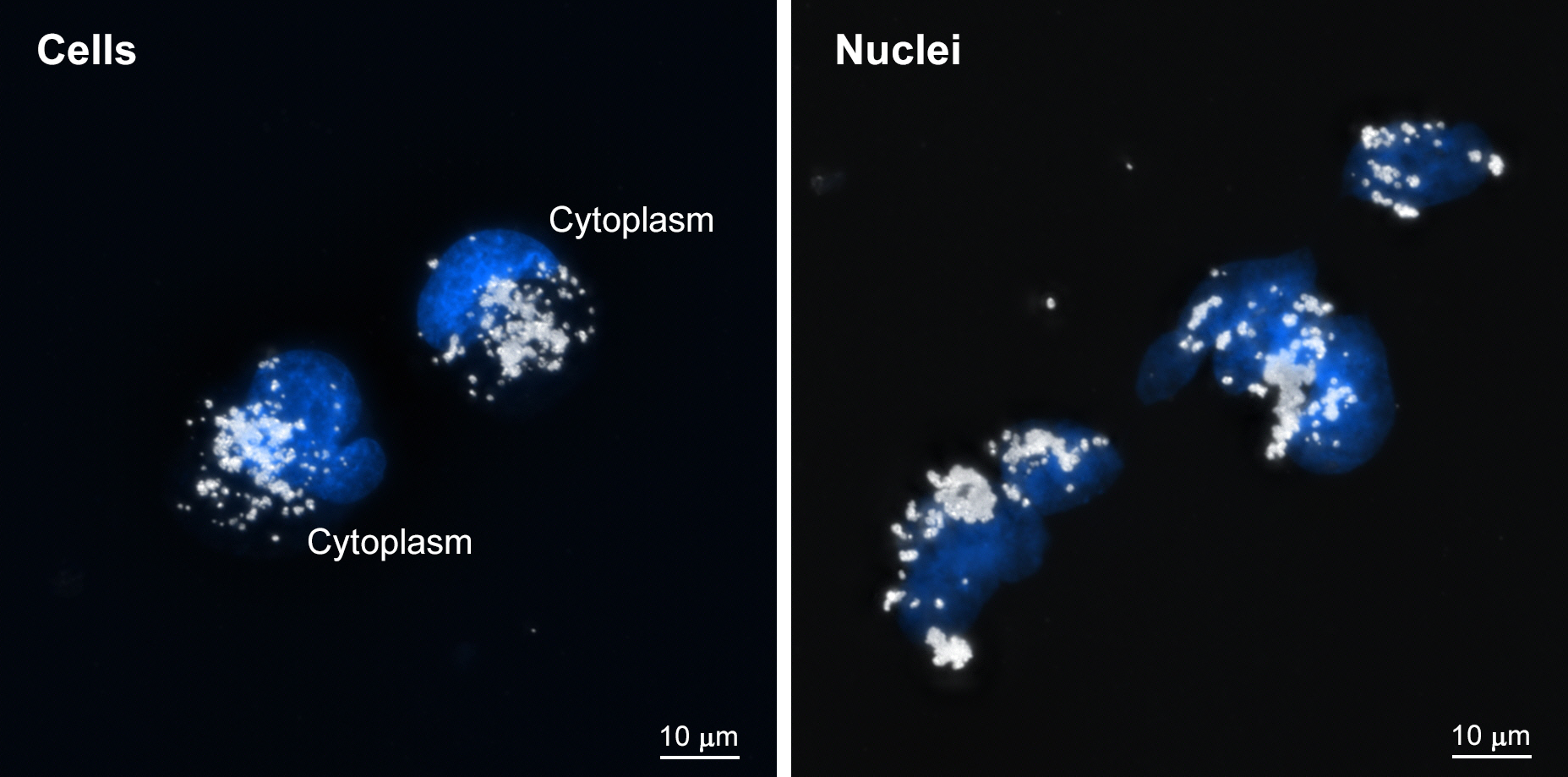

Supplement: S3 Fig — Cells (A) were incubated with 10ug/ml TiO2 Degussa. The image on the left (B) shows the nuclei stained with DAPI surrounded by nanoparticles with dispersed nanoparticles in the cytoplasm. After detergent lysis the cytoplasm is largely removed and some of the nanoparticles are attached to the nuclei. Images were acquired sequentially with fluorescence derived from DAPI stained nuclei (blue) and nanoparticles (white) obtained from with darkfield illumination. The two images were combined using Nikon Elements 5.0. About 30 images of the round cells were taken with Nikon widefield imaging software allowing for the generation of a “Z” stack. The images were sharpened using an extended depth focusing algorithm. (TIF) [file pone.0219078.s003.tif]

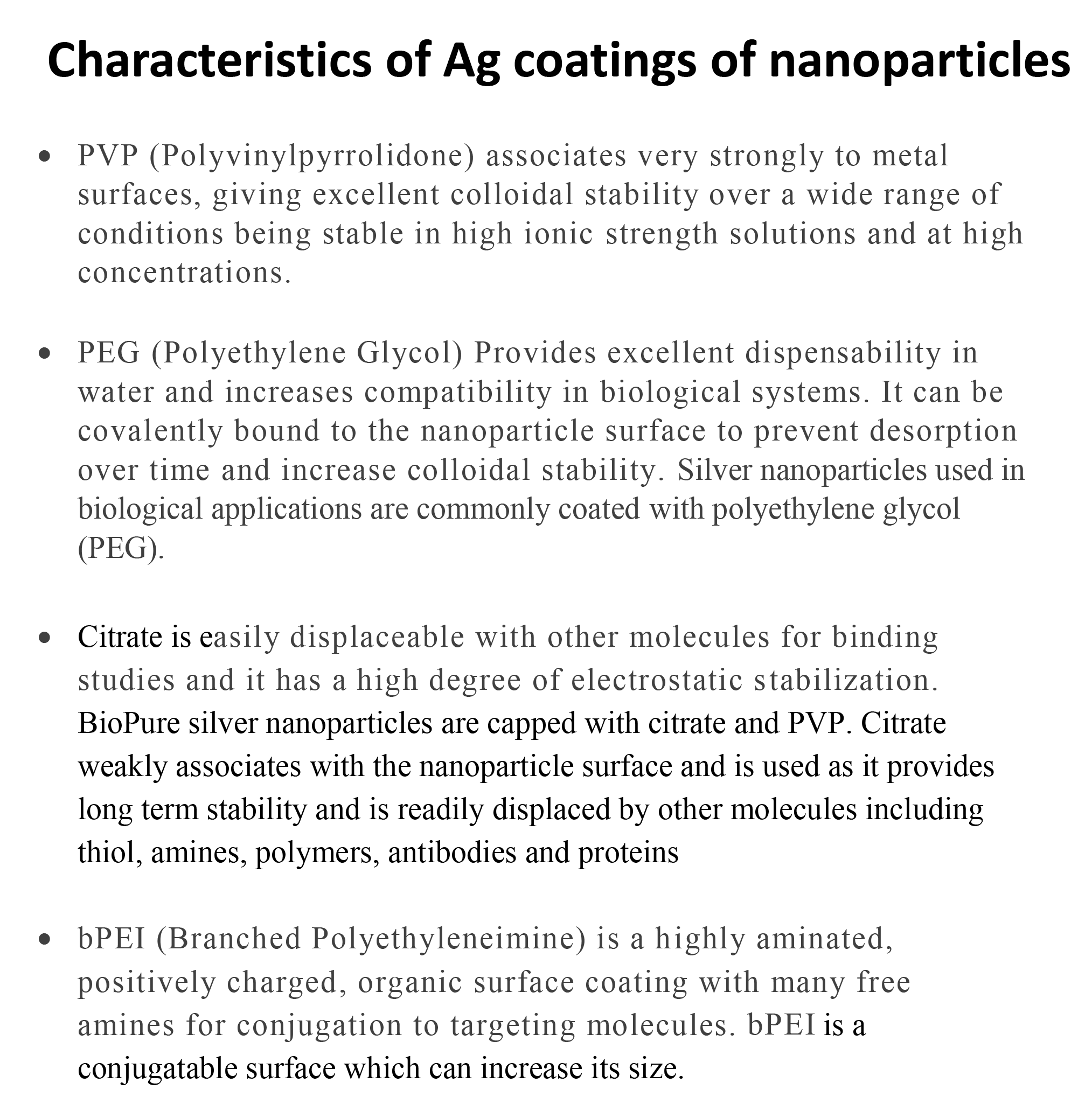

Supplement: S1 Table — Derived from the nanoComposix web site https://nanocomposix.com. (TIF) [file pone.0219078.s004.tif]
